# Carbapenem-Only Combination Therapy against Multi-Drug Resistant *Pseudomonas aeruginosa*: Assessment of In Vitro and In Vivo Efficacy and Mode of Action

**DOI:** 10.3390/antibiotics11111467

**Published:** 2022-10-25

**Authors:** Brendan Mackay, Benjamin J. Parcell, Sally L. Shirran, Peter J. Coote

**Affiliations:** 1 Biomedical Sciences Research Complex, School of Biology, University of St Andrews, The North Haugh, St Andrews, Fife KY16 9ST, UK; 2 NHS Tayside, Medical Microbiology, Ninewells Hospital and Medical School, Dundee DD1 9SY, UK

**Keywords:** antibiotic, resistance, meropenem, doripenem, ertapenem, imipenem, beta-lactams, carbapenemase, Gram-negative bacteria

## Abstract

The aim of the study was to determine the efficacy of carbapenem-only combination treatments derived from four approved drugs (meropenem, doripenem, ertapenem and imipenem) against a MDR strain of *P. aeruginosa* in a *Galleria mellonella* larvae infection model. *G. mellonella* larvae were infected with *P. aeruginosa* NCTC 13437 (carrying the VIM 10 carbapenamase) and the efficacy of the six possible dual, four triple, and one quadruple carbapenem combination(s) were compared to their constituent monotherapies. Four of these combinations showed significantly enhanced survival compared to monotherapies and reduced the bacterial burden inside infected larvae but without complete elimination. Bacteria that survived combination therapy were slower growing, less virulent but with unchanged carbapenem MICs—observations that are consistent with a persister phenotype. In vitro time-kill assays confirmed that the combinations were bactericidal and confirmed that a low number of bacteria survived exposure. Mass spectrometry was used to quantify changes in the concentration of carbapenems in the presence of carbapenemase-carrying *P. aeruginosa*. The rate of degradation of individual carbapenems was altered, and often significantly reduced, when the drugs were in combinations compared with the drugs alone. These differences may account for the enhanced inhibitory effects of the combinations against carbapenem-resistant *P. aeruginosa* and are consistent with a ‘shielding’ hypothesis. In conclusion, carbapenem combinations show promise in combating MDR *P. aeruginosa* and are worthy of additional study and development.

## 1. Introduction

*Pseudomonas aeruginosa* is an opportunistic human pathogen and a major cause of multiple healthcare-associated infections but particularly, hospital-, and ventilator-, acquired pneumonia [1]. Immunocompromised patients in intensive care are most at risk, and infection with *P. aeruginosa* is associated with high morbidity and mortality [2]. *P. aeruginosa* is a successful pathogen because its large genome encodes multiple virulence factors and antibiotic resistance mechanisms [3]. Furthermore, it is Gram-negative and intrinsically resistant to many antibiotics meaning that successful treatment can be difficult [2]. In fact, the incidence of healthcare-associated infections by multi-drug resistant (MDR) strains of *P. aeruginosa* (defined as resistant to three or more classes of antibiotics) is increasing worldwide [4].

Until recently, the broad-spectrum carbapenem class of β-lactam antibiotics have been used extensively to treat MDR *P. aeruginosa* infections. Inevitably, this has contributed to increased incidence of carbapenem-resistant *P. aeruginosa*, meaning that carbapenem monotherapy may no longer be an appropriate, or effective treatment option [5]. For example, the World Health Organisation (WHO) published a list of priority resistant bacterial pathogens in 2017, and carbapenem-resistant *P. aeruginosa* was one of three Gram-negative pathogens described as critical—the highest level of concern—requiring immediate development of new treatment options [6]. In some countries in Southern Europe, the proportions of carbapenem-resistant isolates of *P. aeruginosa* were greater than 60% [7]. Notwithstanding the increased morbidity and mortality due to MDR *P. aeruginosa* infections, there is also a huge economic burden. In the USA, an inpatient with a carbapenem-resistant *P. aeruginosa* infection cost 1.23–1.68 times more than a patient with a sensitive infection representing a 69% increase in costs equating to more than $30,000 [8].

In *P. aeruginosa*, resistance to carbapenems is largely conferred by membrane porin mutations, upregulation of membrane-bound efflux pumps, and the acquisition of carbapenemase enzymes. The outer membrane porin OprD is involved in uptake of carbapenems and deletion or inactivation of *oprD* results in reduced susceptibility to imipenem and meropenem (reviewed in [9]). Efflux pumps belonging to the Resistance Nodulation Division (RND) class, such as MexAB-OprM, efflux β-lactams from the cell and mutations resulting in over-expression of these pumps can result in MDR phenotypes [10]. Carbapenemase enzymes that inactivate carbapenems and confer resistance to nearly all β-lactams have spread globally due to horizontal gene transfer and include the serine β-lactamases such as *Klebsiella pneumoniae* carbapenemase (KPC), metallo-β-lactamases (MBL) such as New Delhi MBL (NDM), Verona integron-encoded MBL (VIM) or imipenemase (IMP) [11].

β-lactam resistance has been addressed in the past by using combination treatments of β-lactam antibiotics with β-lactamase inhibitors (BLIs) that were themselves β-lactam molecules, for example tazobactam, sulbactam and clavulanic acid. However, these dual β-lactam combinations have little efficacy on most carbapenemase-carrying bacteria [12]. To combat carbapenem-resistance in *P. aeruginosa*, a range of new combination treatments consisting of carbapenems with novel non β-lactam BLIs have been approved for use in the USA and Europe. These include, meropenem with vaborbactam and imipenem with relebactam—vaborbactam is a cyclic boronic acid, and relebactam is a member of the diazabycylooctane class [13]. Importantly, whilst these new treatments will target bacteria harbouring serine carbapenemases such as KPC, they do not have notable activity against MBLs conferring carbapenem-resistance such as NDM, VIM or IMP [13]. Due to this gap in treatment options for carbapenem-resistant *P. aeruginosa*, novel therapies are still required.

A potentially novel treatment option for MDR *P. aeruginosa* infections could be β-lactam combination therapy. Presently, β-lactam combinations include a β-lactam antibiotic with a β-lactam that acts as a BLI, for example, amoxicillin and clavulanic acid. However, the combination of different classes of β-lactams, none of which have known BLI activity, could have potential. Earlier research in the 1980’s, prior to the introduction or development of resistance to carbapenems, identified synergistic inhibition of *P. aeruginosa* by combinations of many different β-lactams (reviewed in [14]). However, because MDR Gram-negative pathogens were not a serious problem at this time none of these β-lactam combinations were developed further. Current resistance issues justify additional exploration of the potential of β-lactam combinations particularly because these drugs are generally well tolerated by patients with minimal side-effects. Indeed, recent research in the corresponding authors lab identified two β-lactam combinations (ceftazidime + meropenem and aztreonam + meropenem) that had potent, enhanced efficacy against lethal infection by two strains of carbapenem-resistant *P. aeruginosa* in a *Galleria mellonella* infection model [15]. Notably, the enhanced efficacy of these two β-lactam combinations could not be attributed to more potent inhibition of penicillin binding proteins (PBPs) or inhibition of a broader range of PBPs.

The aim of this study was to evaluate the efficacy of all possible dual, triple, and quadruple carbapenem combinations derived from four approved drugs—meropenem, doripenem, ertapenem and imipenem. Efficacy of each combination therapy in vivo was compared with their constituent monotherapies—(i) against a carbapenem-resistant strain of *P. aeruginosa* in a *Galleria mellonella* larvae infection model, and (ii) in in vitro time-kill assays with the same strain. For the most potent combinations identified, the rate of degradation of each carbapenem alone, and in combination, in the presence of *P. aeruginosa* harbouring the VIM10 MBL, was measured by mass spectrometry to gain insight into the inhibitory action.

## 2. Results

### 2.1. A Carbapenemase-Producing Strain of P. aeruginosa Is Resistant to Four Carbapenem Antibiotics

According to the European Committee on Antimicrobial Susceptibility Testing (EUCAST), resistance to carbapenems is defined as: meropenem > 8 mg/L, doripenem > 2 mg/L and imipenem > 4 mg/L [16]. EUCAST state that ertapenem is not active against *P. aeruginosa* [17]. Therefore, in contrast with the antibiotic-susceptible strain NCTC10662, the *P. aeruginosa* strain harbouring the VIM10 carbapenemase (NCTC13437) displayed resistance to meropenem (MEM), doripenem (DOR), ertapenem (ETP) and imipenem (IPM) as expected (Table 1).

### 2.2. Carbapenem Monotherapy of G. mellonella Larvae Infected with P. aeruginosa NCTC13437 Reveals Antibiotic-Dependent Levels of Efficacy

**Figure 1:**
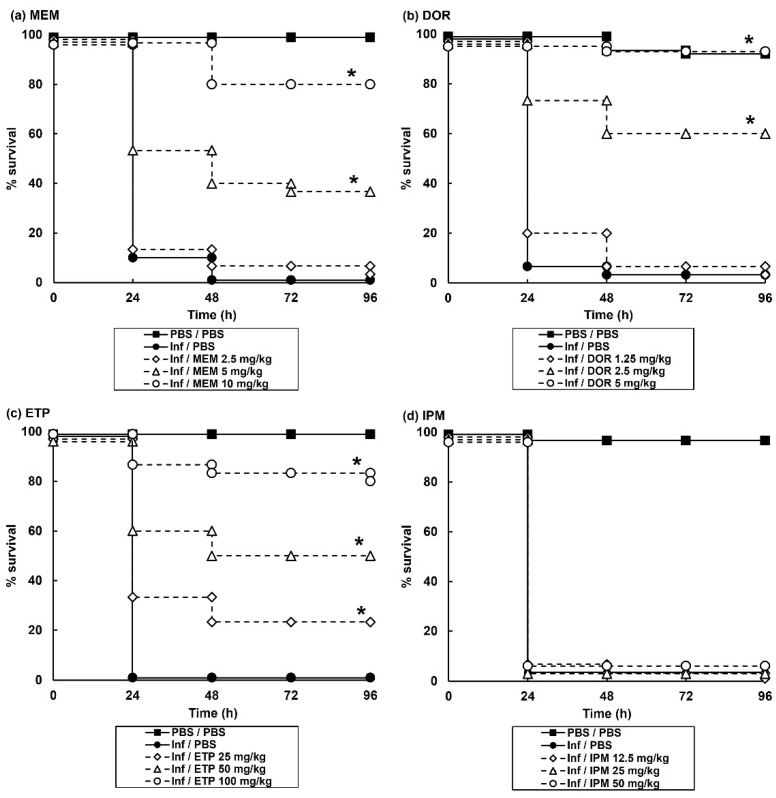
Effect of treatment with carbapenem monotherapies on survival of *G. mellonella* larvae infected with 2.5 × 10^3^ cfu/mL *P. aeruginosa* NCTC13437. Infected larvae were treated with PBS (mock ‘treated’), or: (**a**) MEM (2.5, 5 or 10 mg/kg); (**b**) DOR (1.25, 2.5 or 5 mg/kg); (**c**) ETP (25, 50 or 100 mg/kg); (**d**) IPM (12.5, 25 or 50 mg/kg) and incubated at 37 °C for 96 h. A single dose of the antibiotic treatments was administered 2 h p.i. The uninfected group represents larvae sham-infected with sterile PBS and treated with sterile PBS. * Indicates significantly enhanced survival compared to infected larvae treated with PBS (*p* < 0.05, log rank test with Holm correction for multiple comparisons); *n* = 30 (pooled from duplicate experiments).

Initial experiments determined the efficacy of monotherapy (a single dose, administered 2 h post-infection (p.i) with each of the carbapenem antibiotics on *G. mellonella* larvae infected with a lethal dose (2.5 × 10^3^ cells/mL) of *P. aeruginosa* NCTC13437. Monotherapy with DOR or MEM showed dose-dependent efficacy with doses of 5 mg/kg and 10 mg/kg, respectively, offering high levels of protection 96 h p.i (Figure 1). ETP monotherapy also showed efficacy but only at very high doses of 50 and 100 mg/kg. In contrast, IPM monotherapy of infected larvae provided no protection even at a high dose of 50 mg/kg (Figure 1).

These monotherapy experiments allowed the selection of doses of each individual antibiotic for subsequent study of all possible carbapenem combinations. Doses of each constituent antibiotic that had minimal therapeutic benefit as a monotherapy were selected for combination testing because this allows easy identification of combinations that offer enhanced efficacy compared to their constituent monotherapies.

### 2.3. Treatment of G. mellonella Larvae Infected with P. aeruginosa with Combinations of Carbapenems Results in Enhanced Efficacy Compared to Monotherapies

**Figure 2:**
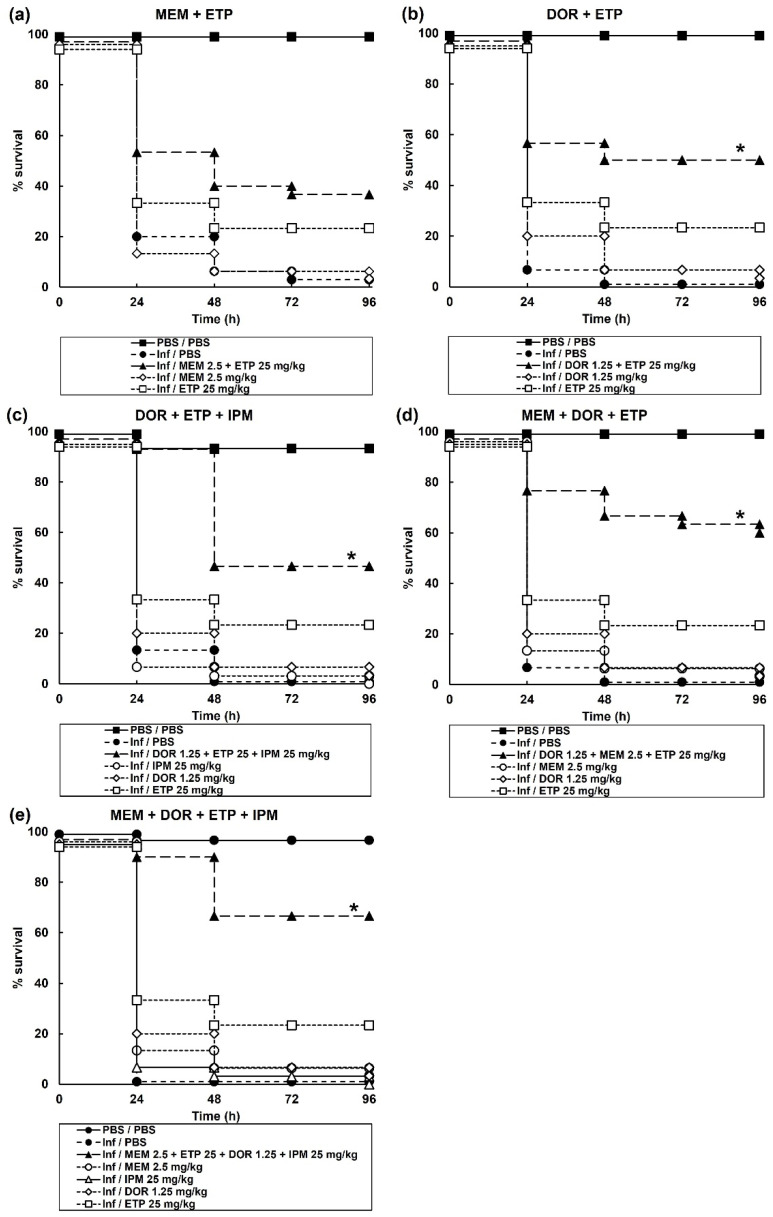
Effect of treatment with carbapenem monotherapies and dual, triple, and one quadruple combination on survival of *G. mellonella* larvae infected with 2.5 × 10^3^ cfu/mL of *P. aeruginosa* NCTC13437. Infected larvae were treated with PBS (mock ‘treated’), carbapenem monotherapies, or carbapenem combinations: (**a**) MEM (2.5 mg/kg) + ETP (25 mg/kg); (**b**) DOR (1.25 mg/kg) + ETP (25 mg/kg); (**c**) DOR (1.25 mg/kg) + ETP (25 mg/kg) + IPM (25 mg/kg); (**d**) MEM (2.5 mg/kg) + DOR (1.25 mg/kg) + ETP (25 mg/kg); or (**e**) MEM (2.5 mg/kg) + DOR (1.25 mg/kg) + ETP (25 mg/kg) + IPM (25 mg/kg). A single dose of the antibiotic treatments was administered 2 h p.i and larvae were incubated at 37 °C for 96 h. The uninfected group represents larvae sham-infected with sterile PBS and treated with sterile PBS. * Indicates significantly enhanced survival compared to each monotherapy alone (*p* < 0.05, log rank test with Holm correction for multiple comparisons); *n* = 30 (pooled from duplicate experiments).

An initial screen of the effect of 6 possible dual, 4 triple, and 1 quadruple carbapenem combination(s) on survival of *G. mellonella* larvae infected with a lethal dose of *P. aeruginosa* NCTC13437 is shown in Table 2. One dual combination, DOR + ETP; two triple combinations, MEM + DOR + ETP and DOR + ETP + IPM, and the single quadruple combination (MEM + DOR + ETP + IPM), showed significantly enhanced survival compared to monotherapies. Following this initial screen, the best combinations were studied in greater detail and confirmed significantly enhanced efficacy of the above combinations compared to sham treatment with either PBS or each constituent monotherapy (Figure 2). In summary, *G. mellonella* larvae infected with a carbapenemase-carrying strain of *P. aeruginosa* were successfully treated with carbapenem combinations.

**Figure 3:**
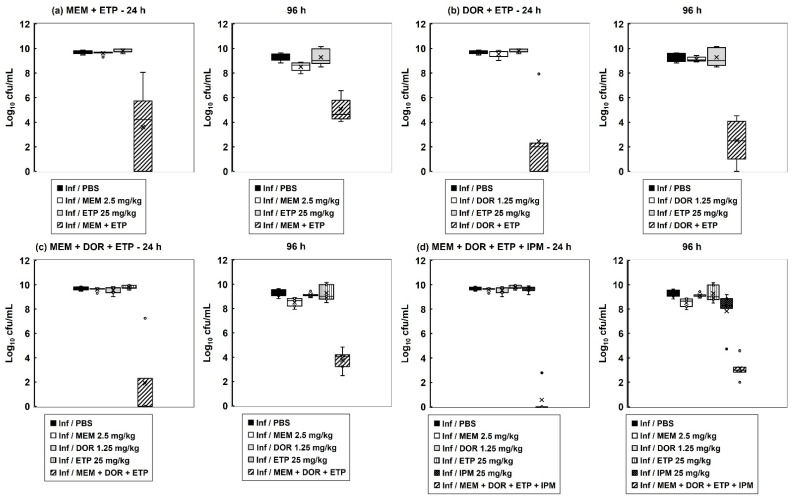
The effect of carbapenem monotherapies and dual, triple, and quadruple carbapenem combinations on the internal burden of *P. aeruginosa* NCTC13437 in *G. mellonella* larvae. Larvae were infected with 2.5 × 10^3^ cfu/mL of *P. aeruginosa* NCTC13437 and treated with either PBS (mock ‘treated’), or a single dose of each carbapenem, or a combination of: (**a**) MEM (2.5 mg/kg) + ETP (25 mg/kg); (**b**) DOR (1.25 mg/kg) + ETP (25 mg/kg); (**c**) MEM (2.5 mg/kg) + DOR (1.25 mg/kg) + ETP (25 mg/kg); (**d**) MEM (2.5 mg/kg) + DOR (1.25 mg/kg) + ETP (25 mg/kg) + IPM (25 mg/kg) at 2 h p.i. Larvae were incubated at 37 °C, and the internal burden of *P. aeruginosa* was determined from five individual larvae per treatment group after 24 and 96 h at 37 °C. The ‘×’ indicates the mean, the bar indicates the median and the error bars show the highest and lowest values within the dataset. Outlier data is shown as independent points. Each combination treatment showed a significant reduction in bacterial burden compared with each monotherapy (*p* < 0.05, the Mann–Whitney *U*-test; *n* = 5).

Correlating with the enhanced survival conferred by carbapenem combination therapy, the internal burden of bacteria within the infected larvae was drastically reduced (Figure 3). The level of reduction in bacterial burden correlated with the degree of enhanced efficacy conferred by each combination treatment tested. With each combination, the number of bacteria recovered from treated larvae was significantly reduced 24 h p.i in comparison to the constituent monotherapies. Notably, 96 h p.i, the reduction in bacterial burden was maintained, but infecting bacteria were never eliminated by any of the combination treatments. For example, the most potent, quadruple carbapenem treatment, that resulted in 67% of infected larvae surviving at 96 h p.i, resulted in an approximate 8-log_10_ reduction in internal bacteria at 24 h p.i but only an approximate 6-log_10_ reduction at 96 h p.i compared with monotherapies. This trend of an initial large reduction in infecting bacteria but the survival of a small population over the duration of the infection was replicated with each of the carbapenem combination treatments (Figure 3).

In conclusion, the carbapenem combination treatments were initially strongly bactericidal but appeared to select a small population of bacteria that remained viable.

### 2.4. The Inhibitory Action of Carbapenem Combinations versus P. aeruginosa Is Bactericidal but Does Not Eliminate All Bacteria In Vitro

**Figure 4:**
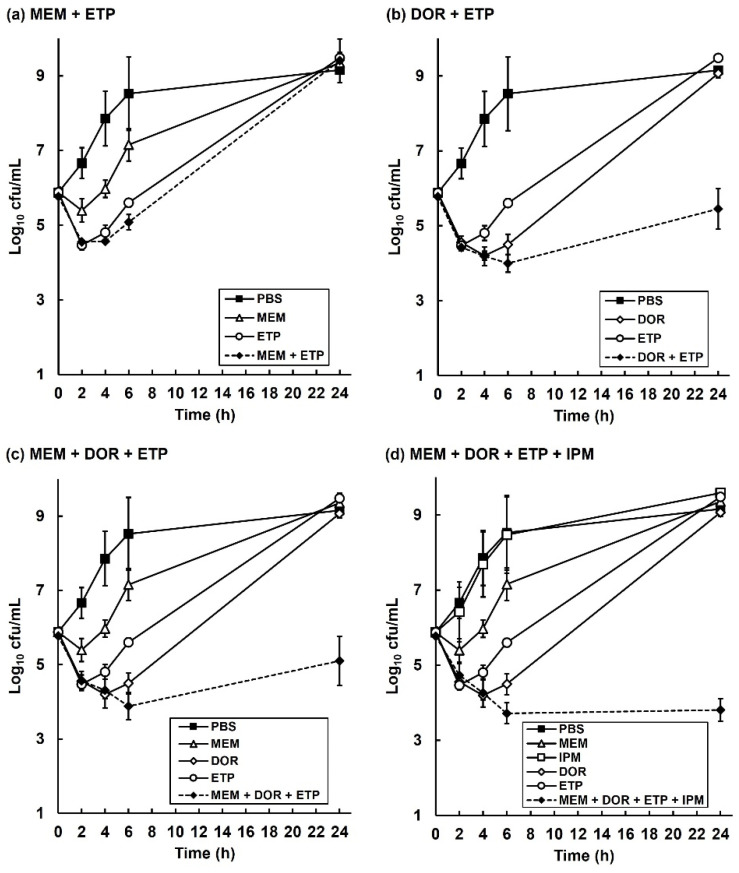
Time-kill assays comparing the effect of exposure to single carbapenems with carbapenem combinations on the growth and viability of *P. aeruginosa* NCTC13437 in vitro. Bacteria were exposed to carbapenem concentrations at MIC_0_._5_ for 24 h at 37 °C in MHB. Combinations tested were: (**a**) MEM (32 mg/L) + ETP (128 mg/L); (**b**) DOR (32 mg/L) + ETP (128 mg/L); (**c**) MEM (32 mg/L) + DOR (32 mg/L) + ETP (128 mg/L); (**d**) MEM (32 mg/L) + DOR (32 mg/L) + ETP (128 mg/L) + IPM (256 mg/L). For each condition tested, viable bacteria were measured after 2, 4, 6 and 24 h exposure. Each experiment was performed in duplicate and the mean ± SEM is shown.

To gain insight into the inhibitory action of the carbapenem combinations, in vitro time-kill assays were done. The effect of exposure to each carbapenem alone (at MIC_50_) and carbapenem combinations (also at MIC_50_ for each drug) on viability of *P. aeruginosa* NCTC13437 at 37 °C over a period of 24 h is shown in Figure 4. A control population, exposed to PBS increased in cell number over the duration of the experiment. Exposure to each carbapenem alone resulted in an initial small loss of viability over the first 4 h, but the bacteria recommenced growth after 6 h, and after 24 h, each population recovered and grew to the same extent as the PBS control. Exposure to the carbapenem combinations resulted in a similar small loss of viability over the first 4 to 6 h exposure, and after 24 h the bacteria recovered, and the population cell number increased in the presence of each combination (except the quadruple; Figure 4d). Notably, the increase in cell numbers for all combinations at 24 h exposure was less than that observed after exposure to the single antibiotic or PBS (excepting the MEM + ETP combination (Figure 4a) that did grow to the same extent after 24 h as the control). The quadruple combination did not recommence growth at all after 24 h and survivors remained low (Figure 4d). Again, the most potent combinations showed an initial bactericidal effect but none of them were able to eliminate all bacteria. This supports the in vivo, internal bacterial burden data where, despite the combinations showing enhanced efficacy, they were unable to eliminate all infecting bacteria (Figure 3).

### 2.5. Surviving P. aeruginosa Cells Isolated from G. mellonella Larvae Exposed to Carbapenem Combination Therapy for 96 h Display a Persister Phenotype

Experiments were done to explore the phenotype of *P. aeruginosa* cells that were recovered from infected *G. mellonella* larvae exposed to a carbapenem combination treatment. Infected larvae were treated with the triple carbapenem combination MEM + DOR + ETP 2 h p.i, and after 96 h incubation, surviving *P. aeruginosa* cells were isolated on PIA from five randomly selected larvae. A single colony from each of these larvae was then resubbed on NA at 37 °C. From these five stock plates, fresh MHB cultures were grown and the growth rate, virulence and the carbapenem MICs of each of these isolates determined. 

**Figure 5:**
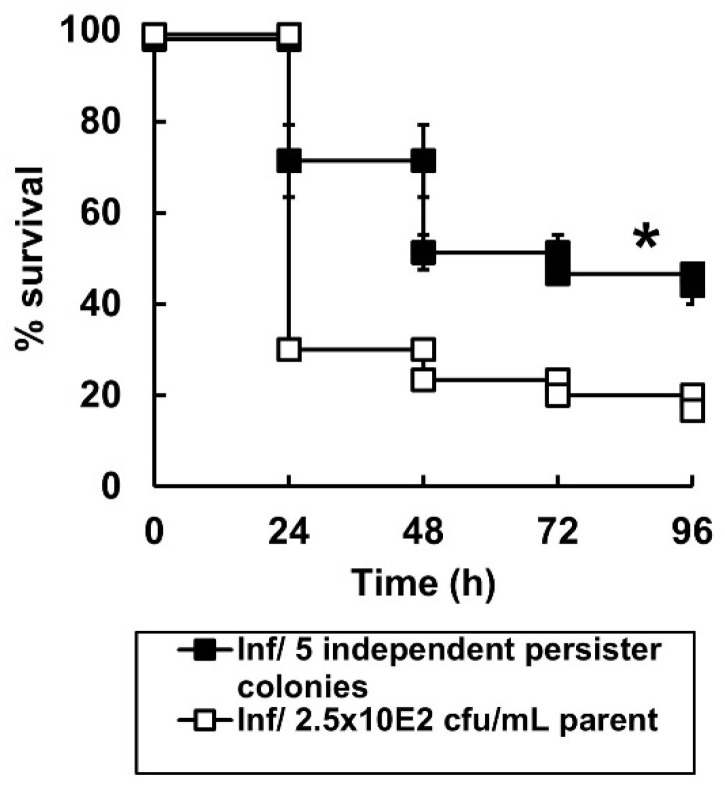
Virulence of untreated *P. aeruginosa* NCTC13437 compared with five persister colonies of the same strain that survived exposure to combination therapy in *G. mellonella*. Groups of *G. mellonella* larvae were infected with 2.5 × 10^2^ cfu/mL of *P. aeruginosa* NCTC13437 not exposed to carbapenem therapy, or, identical numbers of 5 independent persister isolates of the same strain that were isolated from 5 *G. mellonella* larvae that had been infected and treated with the carbapenem combination MEM + DOR + ETP after 96 h. The experiment was carried out in duplicate and the survival curves for the larvae infected with the 5 combination-treated persister isolates were pooled and the mean ± SEM is shown. * Indicates significantly enhanced survival compared to larvae infected with *P. aeruginosa* NCTC13437 not exposed to carbapenem combination therapy (*p* < 0.05, log rank test with Holm correction for multiple comparisons); *n* = 30.

The growth rate of each of the five combination-treated, survivor isolates in MHB at 37 °C was compared to the original, untreated *P. aeruginosa* NCTC13437 and is shown in Table 3. With each isolate the growth rate was approximately half that of the untreated strain. Thus, despite three rounds of replication after their isolation, without any exposure to carbapenems, the ability of these treatment-survivor isolates to replicate optimally was still impaired. Each of the treated isolate cultures were then used to measure virulence in *G. mellonella* and compared with the original, untreated strain (Figure 5). A 10-fold smaller inoculum was used compared with the original efficacy experiments to allow for easier comparison of virulence between the different treatment-survivor isolates and the untreated strain. Furthermore, survival of larvae infected by the five combination-treated isolates was plotted as the mean ± SEM, rather than individual survival lines, to show the difference in virulence more clearly. Notably, the combination-treated isolates were significantly less virulent than the original strain (Figure 5). Again, this observed difference was measured after three additional rounds of replication, without the presence of any carbapenems, since their original isolation from the combination-treated larvae. Finally, the MIC of each of the carbapenems for each of the five treatment-survivor isolates was compared with the untreated parent strain. The MIC values for all isolates were the same as the original parent strain (shown in Table 1). 

In summary, the combination-treatment selected for a sub-population of *P. aeruginosa* cells that did not proliferate in the larvae, are impaired in terms of their ability to replicate and less virulent, but not more resistant to the carbapenems. These observations are consistent with these combination-treatment survivors possessing a persister phenotype. 

### 2.6. The Degradation of Individual Carbapenems in The Presence of Carbapenemase-Carrying P. aeruginosa NCTC13437 Occurs at Different Rates When in Combination with Other Carbapenems Than Alone

**Figure 6:**
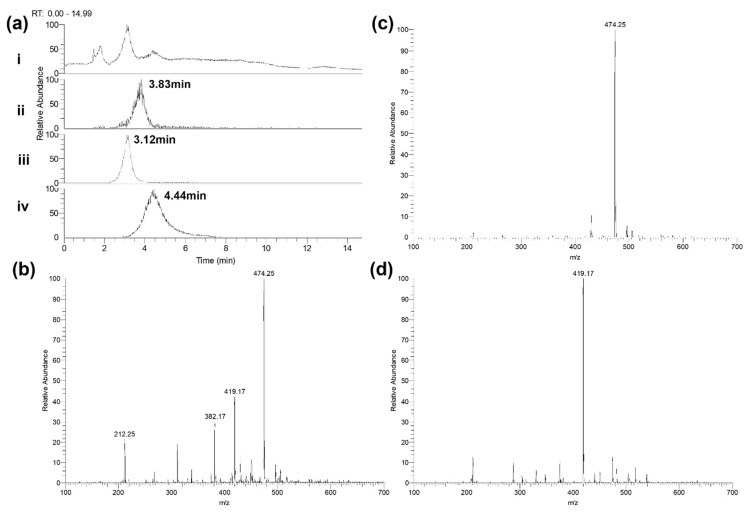
Detection of carbapenems by LC-MS. (**a**) Chromatograms of meropenem, ertapenem and doripenem solutions at 10 mg/L in PBS—(i) total ion chromatogram (TIC); (ii) extracted ion chromatogram (XIC) of meropenem—expected mass 381.5–383.0 m/z; (iii) XIC of ertapenem—expected mass 473.5–475.0 m/z; (iv) XIC of doripenem—expected mass 418.5–420.0 m/z. (**b**) Meropenem—spectrum of elution at 3.83 min. Expected mass of 382.2 m/z. (**c**) Ertapenem—spectrum of elution at 3.12 min. Expected mass of 474.2 m/z. (**d**) Doripenem—spectrum of elution at 4.44 min. Expected mass of 419.2 m/z. Representative data of repeat experiments is shown.

One explanation why carbapenems, with a broadly similar inhibitory action, could be more potent in combination, compared to single drugs alone, versus resistant strains expressing a carbapenemase, is the ‘shielding’ hypothesis. Accordingly, one carbapenem could bind preferentially, or with higher affinity, to the carbapenemase, thus sequestering the hydrolytic capacity of the enzyme, and allowing the other carbapenem(s) in the combination to better inhibit the target PBPs. LC-MS was used to measure the change in concentration of single carbapenems in the presence of *P. aeruginosa* NCTC13437 and this was compared with the change in concentration of the same drug in combination with other carbapenems. Example spectra and chromatograms for each carbapenem are shown (Figure 6). 

**Figure 7:**
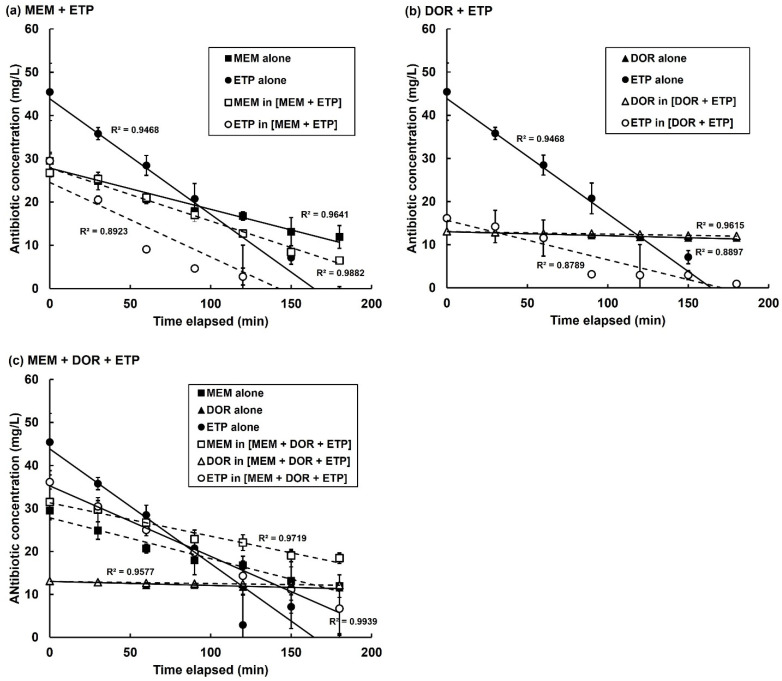
Decline in concentration of individual carbapenems either alone or in combination in the presence of *P. aeruginosa* NCTC13437. Antibiotic concentrations were measured using LC-MS. Carbapenems present in three combination treatments were studied: MEM + ETP, DOR + ETP and MEM + DOR + ETP. Carbapenem concentrations in PBS were—MEM—64 mg/L, DOR—32 mg/L and ETP—256 mg/L. Following this, 2.5 × 10^3^ cfu/mL of *P. aeruginosa* cells, washed and resuspended in PBS, were added and the change in concentration of each carbapenem alone, or in combination, was measured over a period of three hours at 37 °C. The experiment was performed in duplicate and the mean ± SEM is shown. Linear regression with R^2^ values for each condition are shown.

The constituent carbapenems making up three combination treatments were studied: MEM + ETP, DOR + ETP and MEM + DOR + ETP. The change in concentration of each carbapenem alone, or in combination, was measured over a period of three hours at 37 °C in PBS in the presence of 2.5 × 10^3^ cfu/mL of bacteria (Figure 7 and Table 4). The concentration of MEM, DOR or ETP alone declined over the course of the experiments in a linear fashion. Notably, the measured drop in concentration of each carbapenem in the presence of the bacteria occurred at differing rates revealing a hierarchy in susceptibility to degradation. For example, ETP is degraded most rapidly, followed by MEM and lastly DOR. This order of susceptibility correlates with the MIC values of the carbapenems, with the bacteria being most resistant to ETP followed by MEM and DOR (Table 1 and Figure 1).

Study of the rates of degradation of the same carbapenems but in combinations (MEM + ETP, DOR + ETP and MEM + DOR + ETP) revealed significant differences in their rates of decline compared with the drugs alone (Figure 7 and Table 4). With the MEM + ETP combination, the rate of degradation of MEM increased by 34% and ETP decreased by 36% compared with degradation of the drugs alone (Figure 7a; Table 4). This would be consistent with preferential degradation of MEM over ETP and indicate protection, or ‘shielding, of ETP. In contrast, the DOR + ETP combination resulted in a decrease in the rate of degradation of both carbapenems by 37 and 66%, respectively, compared with the drugs alone (Figure 7b). With the triple combination (MEM + DOR + ETP), the rates of degradation of all three carbapenems were reduced by 18, 48 and 39%, respectively, compared with the drugs alone Figure 7c; Table 4). With the combinations that include DOR, despite the rate of DOR degradation decreasing by a significant amount, the degradation rates of this carbapenem are small compared to the other carbapenems and the actual concentration of the drug is only slightly reduced (Figure 7b,c; Table 4). This apparent stability of DOR in the presence of the carbapenemase-carrying *P. aeruginosa*, either alone or in combination, could hide any potential protection the drug confers on MEM or ETP.

In summary, the degradation of individual carbapenems in the presence of carbapenemase-carrying *P. aeruginosa* is altered, and often significantly reduced, when the carbapenems are in combinations compared with the drugs alone. These differences may account for the enhanced inhibitory effects of carbapenem combinations against carbapenem-resistant *P. aeruginosa*.

## 3. Discussion

Increasing prevalence of *P. aeruginosa* carrying MBLs has highlighted a clinical need to find effective treatments for these strains. Recently introduced combination therapies consisting of novel, non-β-lactam β-lactamase inhibitors with a conventional β-lactam antibiotic are not effective against strains expressing MBLs. Some antibiotic combination therapies have been shown to result in improved outcomes, in terms of morbidity and mortality, for high-risk patients compared with monotherapies [18]. Included within these combination therapies are dual β-lactam treatments that were at least as effective as other combinations with less side effects [19]. A specific form of dual β-lactam therapy is dual-carbapenem therapy (DCT) that was first reported as a potential treatment for carbapenem-resistant *Klebsiella pneumoniae* [20]. DCT (meropenem + imipenem) was recently reported to be successful in a murine sepsis model of infection with a carbapenemase-carrying strain of *Acinetobacter baumannii* [21]. In 2013, administration of DCT, in the form of combinations of ertapenem with doripenem or meropenem, cured three patients of infections with carbapenem-resistant *K. pneumoniae* [22]. Since this report, other studies have shown that DCT can be a successful intervention [23,24]. A recent systematic review and meta-analysis of DCT concluded that patient mortality due to infection with carbapenem-resistant *Enterobacterales* was lower compared to controls and was well tolerated, but that more research is required, and a randomised control trial needs to be published [25].

Notably, most of the successful reports of DCT refer to carbapenem-resistant *K. pneumoniae* and we could find none that involve carbapenem-resistant *P. aeruginosa*. In 2020 the UKCPA Pharmacy Infection Network (PIN) recommended for severe carbapenemase-producing *Enterobacterales* infection (including respiratory tract infections,) clinicians should use a minimum of two antibiotics to which the organism is susceptible. They noted that there is insufficient evidence to conclude which combinations are most effective [26]. Clearly further research in this area including *P. aeruginosa* is warranted. The *P. aeruginosa* NCTC13437 strain used in this study carries the VIM10 MBL and the measured MIC values indicated that the strain was resistant to all four carbapenems tested [16]. Notably, the level of resistance varied with very high MIC values for ertapenem and imipenem (both >256 mg/L) and lower MIC values for meropenem (64 mg/L) and doripenem (32–64 mg/L). These differences in the MIC values were reflected in the degree of efficacy of each antibiotic alone versus infected larvae—both meropenem and doripenem showed efficacy, whilst ertapenem was only efficacious at very high doses (100 mg/kg), and imipenem had no therapeutic benefit even at high doses. The efficacy in vivo of meropenem and doripenem, despite having MIC values against *P. aeruginosa* NCTC13437 that indicate it is resistant, supports a recent study that attributed this contradiction to differences in the concentration of free zinc ions (between culture broth and in vivo) that are required for MBLs to function [27]. Standard MHB has a higher concentration of zinc than present in the murine host environment indicating that MBLs would function more effectively in the broth than in vivo thus explaining the differences in bacterial susceptibility. Our results could also be explained by this phenomenon if zinc levels are also lower in *G. mellonella* larvae.

In this study we have shown that carbapenem combination therapy may also represent an effective way to treat infections with *P. aeruginosa* expressing an MBL. The most effective combinations identified were the double combinations of ertapenem with meropenem or doripenem, the triple combination of meropenem + doripenem + ertapenem, and the quadruple treatment (all four tested carbapenems). This is consistent with previous work studying the effectiveness of dual carbapenem combinations that revealed successful therapy with ertapenem plus meropenem or doripenem to treat patients with carbapenemase-carrying *K. pneumoniae* infections [28].

Other dual and triple treatments also showed enhanced efficacy to a lesser extent, but common to all the effective combinations was the presence of ertapenem. Previous in vitro studies identified that dual combinations of carbapenems, many with ertapenem, were synergistic against *K. pneumoniae* strains producing carbapenemases [29,30,31]. Ertapenem was also the most common carbapenem found in previously successful DCT studies against carbapenem-resistant *K. pneumoniae* expressing a KPC carbapenemase [20,32]. Anderson et al. [33] proposed that ertapenem is the least stable and most susceptible carbapenem to hydrolysis by the KPC carbapenemase. This prompted Bulik & Nicolau [20] to hypothesise that the enhanced efficacy of carbapenem combinations could be explained by the KPC carbapenemase preferentially binding and hydrolysing ertapenem thus ‘shielding’ the other more stable carbapenem in the combination from hydrolysis and allowing it to inhibit PBPs with reduced hindrance. Consistent with these findings, in this study utilising LC-MS to measure changes in carbapenem concentrations, ertapenem was the carbapenem that was degraded most rapidly in the presence of *P. aeruginosa* carrying the VIM 10 carbapenemase. Meropenem was degraded more slowly and doripenem was degraded only slightly and was clearly the most stable carbapenem tested. Furthermore, this hierarchy of degradation was consistent with the measured MIC values for the carbapenems with doripenem having the lowest MIC and ertapenem the highest. Supporting the ‘shielding’ hypothesis, when carbapenems were exposed to the VIM-10 carrying *P. aeruginosa* in combinations, their degradation rates were significantly altered in comparison with their rates of degradation alone. For example, with the ertapenem + meropenem combination, the rate of degradation of meropenem was enhanced at the expense of ertapenem suggesting that meropenem was acting to ‘shield’ ertapenem from degradation. This result contradicts many previous observations that suggested ertapenem was preferentially degraded over meropenem (or doripenem). However, these studies were measuring the effect in the presence of the serine-carbapenemase KPC and not the VIM-10 MBL as in this case. Notably, with the ertapenem + doripenem combination the degradation of both antibiotics was reduced—doripenem by just over a third and ertapenem by two-thirds. This also happened with the triple combination of ertapenem + meropenem + doripenem where degradation of all three was reduced. It is difficult to draw a definitive conclusion from this data regarding individual carbapenems ‘shielding’ others, but there are still large reductions in the rate of degradation of individual carbapenems when in combination that are consistent with the ‘shielding’ hypothesis that could account for the enhanced efficacy of these same combinations over monotherapies versus VIM-10 carrying *P. aeruginosa* in vivo. Other than ‘shielding’, an alternative explanation could simply be that in the dual and triple combinations there is an excess of carbapenem substrate meaning that the rate of degradation is slowed compared with exposure to the drugs alone that could also account for the enhanced efficacy of combinations. Additional experimentation will be required to further understand these potential mechanisms.

Despite providing enhanced efficacy, none of the combinations cleared infected larvae of all bacteria—burden assays revealed low numbers of surviving *P. aeruginosa* 96 h p.i after combination therapy (Figure 3). Supporting this, in vitro time-kill assays showed that the combination treatments were bactericidal, but that a small number of bacteria survived after 24 h of exposure to each combination and resumed growth (Figure 4). The surviving bacteria in the treated larvae could represent antibiotic persister populations induced in vivo. Antibiotic persistence has been defined as the ability of a population subset to survive a bactericidal drug concentration [34]. Bacterial persistence has been shown to be induced by nutritional and physical stress (including exposure to antibiotics), dormancy, and reduction in metabolic activity (reviewed in [35]). Isolates of these surviving bacteria randomly selected from treated larvae possessed some characteristics of an antibiotic persister phenotype. For example, after sub-culture without the presence of antibiotics, the MICs of each carbapenem for each of these isolates was the same as the untreated parent strain. Furthermore, the growth rate of these same isolates in MHB was halved, and their pathogenicity versus *G. mellonella* was significantly reduced, compared to the original, untreated parent strain. Thus, the persister isolates did not have increased resistance to carbapenems but displayed ‘reduced fitness’ in terms of slower growth rates and decreased virulence. These phenotypic differences were measured after subculture without exposure to antibiotics implying that heritable changes must have occurred in the original persister population. Persisters have been shown to undergo DNA damage and induction of cellular stress responses, such as the SOS response, that can result in the acquisition of mutations that could explain the heritable changes of reduced growth rate and virulence observed in this work [35].

Evidence of *P. aeruginosa* forming persister cells in vivo has been identified in cystic fibrosis patients undergoing antibiotic treatment for chronic lung infection [36,37]. The role of antibiotic persistence in the relapse of bacterial infections is an important area of research and the relationship between the two is not fully understood. Most studies on antibiotic persistence have been carried out in vitro and do not accurately represent the true nature of persistence during real infection in the presence of a functioning host immune system. The data presented here reveal that *G. mellonella* larvae could be used as an accessible in vivo model to further characterise the role of persistence in infections and antibiotic therapy. Supporting these results, an independent study also identified the formation of antibiotic persister cells of *Acinetobacter baumanii* after exposure to β-lactams in vivo using *G. mellonella* larvae [38].

In summary, this work has shown that carbapenem combination treatments offer enhanced efficacy against infections by an MBL-carrying strain of *P. aeruginosa* compared with monotherapies. The rate of degradation of individual carbapenems in the presence of carbapenemase-carrying *P. aeruginosa* was significantly different when the drugs were in combinations compared with the drugs alone. The reduced degradation of some carbapenems in combination could be explained by preferential degradation by the carbapenemase of one drug over another. This would allow one drug in the combination to be less inhibited and is consistent with a ‘shielding’ mechanism. This could explain why carbapenem combinations were more efficacious than monotherapies. The carbapenem combinations were bactericidal but did not eliminate all bacteria in vitro, or in infected larvae, with small populations surviving. Surviving *P. aeruginosa* isolated in vivo displayed characteristics of a persister phenotype with no enhanced resistance compared to the untreated strain but with reduced virulence and growth rate.

## 4. Materials and Methods

### 4.1. Bacteria and Growth Media

Two strains were used: *P. aeruginosa* NCTC13437, an MDR strain harbouring the VEB-1 extended-spectrum β-lactamase (ESBL) and the VIM-10 MBL, that is resistant to carbapenems and other β-lactam antibiotics [39] and, NCTC10662, an antibiotic sensitivity test control strain (https://www.culturecollections.org.uk/products/bacteria/detail.jsp?refId=NCTC+10662&collection=nctc (accessed on 5 October 2022)). Both strains were grown to stationary phase in Mueller-Hinton broth (MHB; Merck, Darmstadt, Germany) at 37 °C with shaking (at 200 rpm) overnight to prepare inocula for antibiotic efficacy testing in vivo.

### 4.2. Antibiotics and G. mellonella Larvae

All antibiotics were purchased from Sigma–Aldrich Ltd. (Dorset, UK). Concentrated stock solutions of antibiotics were prepared in either sterile deionized water alone (ertapenem (ETP)) or in water with DMSO: meropenem (MEM) 15%, doripenem (DOR) 10% and imipenem (IPM) 25% DMSO. Substocks were all made in deionized water where DMSO was diluted to concentrations that had no effect on growth of the *P. aeruginosa* strains or *G. mellonella* larvae. *G. mellonella* larvae were obtained from UK Waxworms Ltd. (Sheffield, UK).

### 4.3. Antibiotic Susceptibility Testing

Minimum inhibitory concentrations (MICs) of each carbapenem against the two *P. aeruginosa* strains were determined in 96-well microplates as previously described [40]. Briefly, doubling dilutions of each carbapenem were prepared in MHB and subsequently inoculated with 1.0 × 10^6^ cfu/mL of *P. aeruginosa*. Microplates were incubated at 37 °C and the MIC was defined as the carbapenem concentration in the first optically clear well after 24 h.

### 4.4. G. mellonella Infection Model

Efficacy of the carbapenems alone or in combination versus *G. mellonella* larvae infected with *P. aeruginosa* NCTC13437 was exactly as described previously [17]. *G. mellonella* at their final instar larval stage were kept at room temperature in darkness. Larvae weighing within the range of 250 to 350 mg were selected for each experiment to ensure consistency in subsequent drug administration and were used within 1 week of receipt. Briefly, groups of 15 larvae were infected with an inoculum of 2.5 × 10^3^ cfu/mL of *P. aeruginosa* cells (unless otherwise stated). Treatment with a single dose of each carbapenem alone, or combinations of carbapenems, were administered 2 h post-infection (p.i). The experiments were repeated in duplicate using larvae from a different batch and the data from these replicate experiments were pooled to give *n* = 30. Survival data were plotted using the Kaplan–Meier method [41] and comparisons made between groups using the log-rank test [42]. In all comparisons with the negative control, it was the uninfected control (rather than the unmanipulated control) that was used and *p* ≤ 0.05 was considered significant. Bacterial burden within larvae from each treatment group was measured exactly as described previously [43,44]. Groups of 30 larvae were infected with *P. aeruginosa* NCTC13437 using the same inoculum sizes as described above. Treatments of carbapenems alone, or combinations of carbapenems, were administered at 2 h p.i. Larvae were incubated in Petri dishes at 37 °C. At 24 h and 96 h p.i, five larvae were randomly selected from each treatment group and surface decontaminated and anaesthetised by washing in absolute ethanol. Each larva was then placed in an Eppendorf tube containing 1 mL of sterile PBS and homogenised using a sterile pestle. Bacterial burden from individual caterpillars was then determined by serial dilution of the homogenate in MHB and plating on Pseudomonas Isolation Agar (Sigma–Aldrich Ltd., Dorset, UK). The detection limit for this assay was 100 cfu/mL of larval homogenate.

### 4.5. Time-Kill Assay

Approximately 1.0 × 10^6^ cfu/mL of *P. aeruginosa* NCTC13437 cells were exposed to PBS (control), carbapenems alone or appropriate combinations of carbapenems in MHB at 37 °C. All carbapenems, either alone or in combinations, were used at concentrations that represented MIC_0_._5_—MEM—32 mg/L, DOR—32 mg/L, ETP—128 mg/L and IPM—256 mg/L. Samples were removed for enumeration of viable bacteria after 2, 4, 6 and 24 h exposure. An initial inoculum was also enumerated as the starting cell number with no exposure to any treatments. Samples were 10-fold serially diluted in MHB prior to plating on Nutrient Agar (NA) plates (Formedium Ltd., Hunstanton, UK). Plates were incubated at 37 °C overnight prior to counting colonies. Each experiment was performed in duplicate and the mean ± standard error of the mean (SEM) plotted.

### 4.6. Isolation and Characterisation of In Vivo Persister Cells

Larvae infected with *P. aeruginosa* NCTC13437 and treated with a single dose of the carbapenem combination MEM + DOR + ETP were left for 96 h. After this time, 5 surviving larvae were randomly selected and infecting *P. aeruginosa* were isolated on PIA as described above. A single colony was randomly picked from an agar plate with the surviving bacteria recovered from each larva and re-subbed onto NA. These five isolates were then sub-cultured again in MHB with shaking at 37 °C and their growth rate, MICs and virulence compared with a control culture of *P. aeruginosa* NCTC13437 that had not been exposed to the triple carbapenem therapy in vivo. To measure growth rate, fresh MHB in 100 mL conical flasks was inoculated from overnight cultures of each of the combination-treated isolates and the untreated control to give a starting optical density (600 nm) of 0.1. These cultures were incubated at 37 °C with shaking and the change in optical density of each culture was measured every 30 min. The experiment was performed in duplicate, and the growth rate of each culture was calculated over the exponential growth part of the curve and calculated as the mean growth rate ± SEM. From the same overnight cultures, MICs of each of the 5 combination-treated isolates and the untreated control culture were measured exactly as described above. Virulence of each of the 5 combination-treated isolates and the untreated control culture was measured in *G. mellonella* larvae as described above with minor modification. Briefly, groups of larvae were infected with an inoculum of 2.5 × 10^2^ cfu/mL of *P. aeruginosa* from overnight MHB cultures. A smaller inoculum was used to infect the larvae compared with the carbapenem efficacy experiments described previously to allow better discrimination of any changes in the degree of virulence between the different *P. aeruginosa* isolates. Survival was determined at 37 °C over 96 h as before.

### 4.7. Quantification of Changes in Carbapenem Concentration in the Presence of P. aeruginosa NCTC13437 by Mass Spectrometry

Quantification of changes in carbapenem concentration in the presence of *P. aeruginosa* NCTC13437 were carried out using liquid chromatography with mass spectrometry (LC-MS) based on the method of [45]. Carbapenems were measured and quantified on a ThermoScientific Ultimate u3000 LC and LCQFleet MS. Calibration curves were plotted of known concentrations of each carbapenem in PBS against the calculated area under the curve for each extracted ion chromatogram (XIC) for m/z value [M-H] from each carbapenem (Supplementary Figure S1). The change in concentration of each carbapenem alone, or in selected combinations, in the presence of *P. aeruginosa* NCCT13437 was then measured. Solutions of each carbapenem alone or combinations were made in PBS using the MIC concentrations of each antibiotic—MEM—64 mg/L, DOR—32 mg/L; ETP—256 mg/L and IPM—512 mg/L. Following this, 2.5 × 10^3^ cfu/mL of *P. aeruginosa* cells, washed and resuspended in PBS, were added to make final reaction volumes of 400 µL. The solutions were mixed, incubated at 37 °C, and 30 µL samples drawn from each vessel every thirty minutes for 3 h post-addition of bacteria. Immediately upon removal from the reaction vessel, all samples were rapidly frozen at −70 °C for later analysis by LC-MS. LC-MS was carried out on a ThermoScientific LCQ Fleet ion trap mass spectrometer with Ultimate u3000 HPLC. 10 µL of sample was injected onto a waters Acquity BEH amide column (2.1 mm × 150 mm). The solvent system consisted of Eluent A: 50% methanol, 50% water; Eluent B: 100% methanol at 0.2 mL/min. The column was equilibrated in 100% eluent B prior to analysis and a blank injection carried out. After injection, the gradient was changed from 100% eluent B to 30% over 7 min, before returning to 100% in 0.1 min and re-equilibrating for a further 7 min. The eluent from the column was sprayed directly into the mass spectrometer and data was collected from 100–1000 m/z in negative ionisation ESI for the duration of the 15 min LC run. From the resultant chromatograms, the area under the curve for each peak (MA) representing each carbapenem was calculated and converted into antibiotic concentration via the calibration curves previously generated. A blank sample of pure methanol was used to flush the system before and after each condition was run. All experiments were performed in duplicate.

## Figures and Tables

**Table 1:** Minimum inhibitory concentration (MIC) of *P. aeruginosa* strains NCTC10662, an antibiotic susceptible control strain, and NCTC13437, an antibiotic-resistant strain carrying the extended-spectrum β-lactamase (ESBL) VEB1, and the carbapenemase VIM10. The experiment was performed in triplicate. MEM—meropenem, IPM—imipenem, DOR—doripenem, ETP—ertapenem.

		MIC (mg/L)			
Strain	Resistance Mechanism	MEM	DOR	ETP	IPM
*P. aeruginosa* NCTC10662	None	2	1	16	8
*P. aeruginosa* NCTC13437	VEB1 and VIM10	64	32–64	>256	>256

**Table 2:** Screen of the efficacy of 6 dual, 4 triple and 1 quadruple carbapenem combination treatments against *G. mellonella* larvae infected with a lethal dose of *P. aeruginosa* NCTC13437. One dose of each monotherapy, dual, triple, or quadruple combination was administered 2 h post-infection (p.i) and survival measured 96 h p.i. * indicates significantly enhanced survival compared to PBS treatment (*p* < 0.05, log-rank test). Most potent combinations are underlined. *n* = 30.

Therapy	Antibiotic(s) or PBS Control	Dose (mg/kg)	% Survival In Vivo 96 h p.i
**Sham** **treatment**	PBS	10 μL PBS	0
**Monotherapy**	MEM	2.5	3.3
	DOR	1.25	3.3
	ETP	25	23.3
	IPM	25	0
**Dual combination** **therapy**	MEM + DOR	2.5 + 1.25	0
	MEM + ETP	2.5 + 25	36.7
	MEM + IPM	2.5 + 25	5
	DOR + ETP	1.25 + 25	50 *
	DOR + IPM	1.25 + 25	13.3
	ETP + IPM	25 + 25	0
**Triple combination** **therapy**	MEM + DOR +ETP	2.5 + 1.25 + 25	60 *
	MEM + DOR + IPM	2.5 + 1.25 + 25	0
	MEM + ETP + IPM	2.5 + 25 + 25	26.7
	DOR + ETP + IPM	1.25 + 25 + 25	46.7 *
**Quadruple combination** **therapy**	MEM + DOR + ETP + IPM	2.5 + 1.25 + 25 + 25	66.7 *

**Table 3:** Growth rates of the *P. aeruginosa* NCTC13347 parent strain, and cultures of 5 random persister colonies. Persisters were isolated after 96 h post-infection from infected *G. mellonella* larvae treated with a single dose of the triple combination of MEM + DOR + ETP. Bacterial growth was measured in MHB at 37 °C with shaking and was performed in duplicate and ± represents the SEM. MEM—meropenem, DOR—doripenem, ETP—ertapenem.

*Pseudomonas aeruginosa* NCTC13437	Growth Rate (Optical Density 600 nm/h)	% Reduction in Growth Rate
Parent strain	0.73 ± 0.17	N/A
Persister colony 1	0.34 ± 0.08	53
Persister colony 2	0.39 ± 0.03	47
Persister colony 3	0.33 ± 0.01	55
Persister colony 4	0.39 ± 0.05	47
Persister colony 5	0.38 ± 0.24	48

**Table 4:** Rate of degradation of MEM, DOR and ETP alone and in combinations in the presence of *P. aeruginosa* NCTC13437 in PBS at 37 °C. Antibiotic concentration was measured using mass spectrometry. Rates of antibiotic degradation were calculated from the linear regression of the mean of two independent experiments showing the change in antibiotic concentration over 180 min at 37 °C. Black shading = the rate of degradation of an antibiotic was increased in combination; grey shading = the rate of degradation of an antibiotic was decreased in combination. MEM—meropenem, DOR—doripenem, ETP—ertapenem.

Carbapenem Treatment	Rate of Carbapenem Degradation (mg/L min^−1^)			Change in Rate of Carbapenem Degradation (%)		
	MEM	DOR	ETP	MEM	DOR	ETP
Carbapenem alone	−0.0953	−0.0092	−0.2671	-	-	-
MEM + ETP	−0.1275	N/A	−0.1722	+34	N/A	−36
DOR + ETP	N/A	−0.0058	−0.0914	N/A	−37	−66
MEM + DOR + ETP	−0.0777	−0.0048	−0.1642	−18	−48	−39

## Data Availability

Data can be made available by the corresponding author on request.

## Supplementary Materials

The following supporting information can be downloaded at: https://www.mdpi.com/article/10.3390/antibiotics11111467/s1, Figure S1: Calibrations of carbapenem concentration versus area under the curve for each LC-MS extracted ion chromatogram.
