# Supplementary material for: Carbapenem-Only Combination Therapy against Multi-Drug Resistant Pseudomonas aeruginosa: Assessment of In Vitro and In Vivo Efficacy and Mode of Action
Source: Antibiotics (Basel). 2022 Oct 25;11(11):1467. doi: 10.3390/antibiotics11111467 (PMC9686798; doi:10.3390/antibiotics11111467)
Supplement: Supplementary file 1 [file antibiotics-11-01467-s001.zip › antibiotics-1983903-supplementary.pdf]

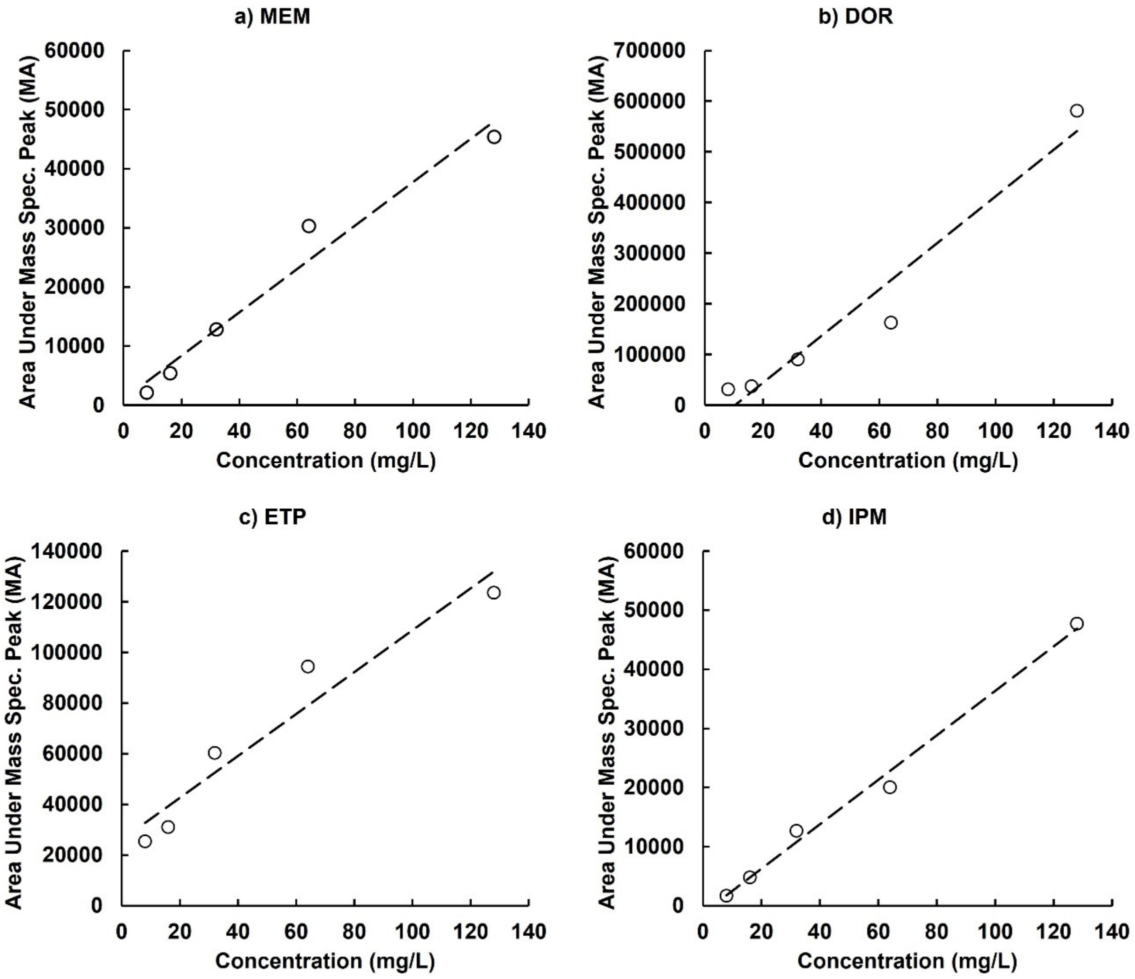

Figure S1. Standards with known concentration (8 – 128 mg/L) of each carbapenem in PBS were analysed by LC-MS and the area under the curve for each extracted ion chromatogram (XIC) for m/z value [M-H] from each carbapenem concentration was calculated and calibration curves plotted.
